# Non typable-*Haemophilus influenzae* biofilm formation and acute otitis media

**DOI:** 10.1186/1471-2334-14-400

**Published:** 2014-07-19

**Authors:** Assaf Mizrahi, Robert Cohen, Emmanuelle Varon, Stephane Bonacorsi, Stephane Bechet, Claire Poyart, Corinne Levy, Josette Raymond

**Affiliations:** 1 Université Paris Descartes, Hôpital Cochin, Bactériologie, 27 rue du Faubourg Saint Jacques, 75679 Paris cedex 14, France; 2 ACTIV, Saint Maur des Fossés, Paris, France; 3 Université Paris Descartes, Hôpital Georges Pompidou, Bactériologie, Paris, France; 4 Université Diderot, Hôpital Robert Debré, Bactériologie, Paris, France

**Keywords:** *Haemophilus influenzae*, Biofilm, AOM, Conjunctivitis

## Abstract

**Background:**

Non-typable *Haemophilus influenzae* (NT-*Hi*) infection is frequently associated with acute otitis media (AOM) treatment failure, recurrence or chronic otitis media. Persistence of otopathogens in a biofilm-structured community was implicated in these situations. Here, we compared biofilm production by *H. influenzae* strains obtained by culture of middle ear fluid (MEF) from children with AOM treatment failure and by strains isolated from nasopharyngeal (NP) samples from healthy children or those with AOM (first episode or recurrence). We aimed to evaluate an association of clinical signs and *in vitro* biofilm formation and establish risk factors of carrying a biofilm-producing strain.

**Methods:**

We used a modification of the microtiter plate assay with crystal violet staining to compare biofilm production by 216 *H. influenzae* strains: 41 in MEF from children with AOM treatment failure (group MEF), 43 in NP samples from healthy children (NP group 1), 88 in NP samples from children with a first AOM episode (NP group 2, n = 43) or recurrent (NP group 3, n = 45) and 44 in NP samples from children with AOM associated with conjunctivitis (NP group 4).

**Results:**

At all, 106/216 (49%) *H. influenzae* strains produced biofilm as did 26/43 (60.5%) in NP samples from healthy children. Biofilm production in MEF samples and NP samples did not significantly differ (40.5% *vs* 60.5%, 55.8%, 56.8% and 31.1% for NP groups 1, 2, 3 and 4, respectively). On multivariate analysis, only presence of conjunctivitis was significantly associated with low biofilm production (OR = 0.3, CI [0.16-0.60], *p* = 0.001). The ampicillin resistance of *H. influenzae* produced by penicillin-binding protein modification was significantly associated with low biofilm production (*p* = 0.029).

**Conclusion:**

We found no association of biofilm production and AOM treatment failure or recurrence. Biofilm production was low from *H. influenzae* strains associated with conjunctivitis-otitis syndrome and from strains with modified penicillin-binding protein.

## Background

Non-typable *H. influenzae* (NT-*Hi*) is a commensal bacterium of the human respiratory tract and can be responsible for non-invasive diseases such as acute otitis media (AOM) and sinusitis. With the expanded use of 7-valent pneumococcal vaccination, *Streptococcus pneumoniae* and NT-*Hi* are the two most common bacteria implicated in AOM [1,2]. Couloigner et al. recently reported that after the 7-valent Pneumococcal Conjugate Vaccine implementation in France, *S. pneumoniae* and NT-*Hi* infection were equally frequent among children with AOM treatment failure. Indeed, the serotype 19A, not included in the vaccine, was the main *S. pneumoniae* serotype reported and represented 84.5% of all serotypes detected [3].

NT-*Hi* is frequently associated with AOM treatment failure, recurrence and otitis media effusion [4,5]. Faden *et al.* demonstrated that nasopharyngeal (NP) colonization with NT-*Hi* is an important risk factor for AOM [6]. AOM is more likely to develop in children with than without frequent NP colonization with NT-*Hi*[6,7]. Kaur *et al.* used multi-locus sequence typing to compare strains isolated from NP and middle ear fluid (MEF) in 34 children during an AOM episode. They found the same sequence type of NT-*Hi* in 31 (84%) children, which highlights the close relationship between strains isolated from both sites [8]. Furthermore, because of the pain caused by tympanocentesis, bacteriological samples of MEF are generally not recommended in most guidelines for AOM followed by paediatricians or general practitioners, except in case of treatment failure [8].

Several studies have shown that NT-*Hi* forms biofilms *in vivo* and *in vitro*[9,10]. A biofilm is a community of microorganisms adhering to a surface and enclosed in a self-produced extracellular matrix (ECM). This structure protects against external aggression such as the host immune system and antibiotics treatment [11]. Biofilm formation was originally suggested to explain the failure to culture NT-*Hi* from middle-ear effusions, resistance to antibiotics and pathogenic behavior. Bacterial biofilms are mainly present in chronic infections, such as chronic pulmonary infections caused by *Pseudomonas aeruginosa* in cystic fibrosis patients or in medical-device–related infections mainly due to *Staphylococcus aureus* or *Staphylococcus epidermidis*[12,13]. Persistence of NT-*Hi* in a biofilm-structured community was implicated in the pathogenesis of chronic and recurrent otitis media [10,11]. The mechanism seems to be an inefficient clearance of bacteria from the middle ear [14]. However, this hypothesis remains controversial [15].

Here, we aimed to determine whether biofilm production is increased in bacterial strains from children with AOM treatment failure. We compared the *in vitro* biofilm-forming ability of *H. influenzae* strains in MEF from children with AOM treatment failure and in NP samples from children with a first episode or recurrent AOM with or without conjunctivitis or in NP microbiota from healthy children. In addition, we evaluated a possible association of clinical signs and *in vitro* biofilm formation and identified risk factors of carrying a *H. influenzae* strain producing biofilm.

## Methods

### A) Patients

After the implementation of PCV7 in France, we conducted two studies in parallel during the same period (May 2007 to April 2009). In the first study, ear, nose and throat specialists obtained MEF samples from children with AOM treatment failure [3]. This study enrolled 143 children (mean age 16.9 ± 9 months). The second study examined the NP carriage of *S. pneumoniae* and *H. influenzae* in healthy children and children with AOM [3].

Treatment failure was defined as otorrhea or bulging of the tympanic membrane, together with fever and otalgia (or its equivalent: irritable or ill-tempered child), despite at least 48 hr of antibiotics, or recurring < 4 days after the end of antimicrobial treatment. Recurrence was defined by the reappearance of AOM signs and symptoms 4 to 30 days after the end of antimicrobial treatment [16]. The definitions of recurrence and failures applied to all patients of all groups.

#### Establishment of patient groups

In the first study of 143 children with AOM treatment failure or recurrence, *H. influenzae* was found in MEF cultures from 45 children, which constituted the "MEF group" in this study.

Among the children enrolled in the NP surveillance study, 98 healthy children and 481 children with AOM were carrying *H. influenzae.* Because tympanocentesis is not recommended for first-line treatment of AOM, we used NP samples and stringent criteria for the diagnosis of otitis. We defined 4 NP groups: 1) NP group 1, *H. influenzae* strains in NP samples from 98 healthy children; 2) NP group 2, *H. influenzae* strains in NP samples from 82 children with a first AOM without conjunctivitis; 3) NP group 3, *H. influenzae* strains in NP samples from 57 children with recurrent AOM without conjunctivitis; and 4) NP group 4, *H. influenzae* strains in NP samples from 342 children with first or recurrent AOM associated with conjunctivitis.

**Figure 1:**
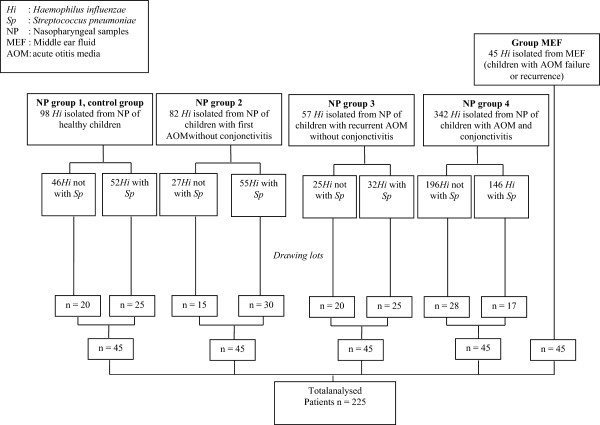
Composition of the groups of children and isolated bacteria from middle ear fluid (MEF) or nasopharyngeal samples (NP).

From each of these 4 groups, we randomly selected representative samples of 45 children (one strain per child). For NP group 4 (first or recurrent AOM), we did not have information about history of otitis for 23 children; 13 had previous otitis, and AOM was the first episode for 9 children. Therefore, because of the low number of children in each subgroup (first or recurrent otitis), this group cannot be divided. Co-carriage of *H. influenzae* and *S. pneumoniae* in NP samples was considered (Figure 1).

#### Ethics approval

The two studies used standardized protocols and common inclusion criteria for AOM and enrolled children of the same age. Furthermore, they involved mostly the same investigators and centralized microbiology laboratories. The protocols were approved by the Saint Germain en Laye Hospital Ethics Committee. Written informed consent was obtained from parents or legal representatives.

### B) Methods

#### Samples

Deep NP samples were taken transnasally with use of a flexible, sterile, soft rayon swab tip. After sampling, swabs were immediately inoculated in transport medium (Copan Venturi Transystem, Brescia, Italy), stored at ambient temperature and sent most often during the day and no more than 48 hr later to the "Centre National de Référence des Pneumocoques" (Hôpital Européen Georges Pompidou, Paris) and to the Robert Debré Hospital Bacteriological Laboratory (Paris). Bacterial strains were frozen in each laboratory at -80°C in brain heart infusion broth (BHI) with 10% glycerol (Mérieux, Marcy l'Etoile, France). MEF was obtained by tympanocentesis and/or by sampling spontaneous discharge according to the recommended clinical practice guidelines. After collection, MEF specimens were transported to the centralized microbiology laboratories.

#### Culture

Swabs and MEF were plated within 24 hr onto chocolate and blood agar (Biomérieux, La Balme Les Grottes, France) and incubated at 37°C for 48 hr with 5% CO_2_. Both *S. pneumoniae* and *H. influenzae* were identified. Isolates of *H. influenzae* were identified by colony morphology and conventional methods of determination. Then, isolates underwent capsular serotyping by the slide agglutination method with specific antisera (Phadebact, Boule Diagnostic, Huddinge, Sweden). The production of ß-lactamase was assessed by a chromogenic cephalosporin test (Nitrocefin, Cefinase; Biomerieux, Marcy l’Etoile, France). Ampicillin-resistant, non-β-lactamase–producing *H. influenzae* (BLNAR) was identified on a 30-μg Cefalotin disk (<17-mm inhibition zone diameter) on *Haemophilus* test medium (HTM) plates (Becton Dickinson, Le Pont-De-Claix, France) according to the 2013 statement of the "Comité de l'Antibiogramme de la Societé Francaise de Microbiologie" [17].

#### Microtiter biofilm formation assay

The biofilm formation by *H. influenzae* isolates was evaluated by a modified microtiter plate assay with crystal violet (CV) staining [18]. This assay was based on the ability of bacteria to adhere to solid polystyrene surfaces by producing a biofilm. Indeed, as a cationic dye, CV will bind to the predominant matrix components (e.g., exopolysaccharides), specifically uronic acids or ketal-linked pyruvate [19]. *H. influenzae* strains were sub-cultured on chocolate agar (Biomérieux, La Balme Les Grottes, France) and incubated at 37°C for 24 hr with 5% CO_2_. Identification of all strains was confirmed by matrix-assisted laser desorption ionization-time of flight mass spectrometry (MALDI-TOF MS, Bruker Daltonics, Germany) with the database provided by the *Haemophilus* National Reference Center [20].

Then *H. influenzae* isolates were grown overnight in BHI broth (Becton Dickinson, Le Pont-De-Claix, France) supplemented with 15 mg/L nicotinamide adenine dinucleotide and hemin (HTM supplemented, Oxoid, Basingstoke Hampshire, UK). The suspension was washed with sterile phosphate buffered saline and diluted 1:200 in fresh supplemented BHI broth. In total, 200 μl *H. influenzae* suspension was inoculated into wells of a polystyrene flat-bottomed 96-well microtiter plates (Nunc, Kracker Scientific, Inc, Albany, New York), which were incubated at 37°C for 24 hr aerobically with 5% CO_2_. The growth of *H. influenzae* was assessed by measuring optical density absorbance at 595 nm (OD_595nm_). Culture media including unattached bacteria was decanted from wells, and the remaining planktonic *H. influenzae* cells were removed by rinsing with distilled water. The wells were air-dried and adhered bacteria were stained with 0.5% (w/v) CV solution (Sigma-aldrich, USA) for 15 min. After rinsing with distilled water, bound CV was released from *H. influenzae* cells by a 20% acetone–80% ethanol solution. Thus, biofilm formation could be measured on both bottoms and sides of wells. Biofilm formation was quantified by measuring the absorbance at OD_595nm_.

A strain *of S. aureus* producing biofilm and a strain of *Lactococcus lactis* non-producing biofilm were included in each experiment as controls. These strains were kindly provided by the Streptococci national reference center (Cochin Hospital, Paris). The assays were performed in triplicate. For each assay, the BHI broth alone was tested to calculate the biofilm formation index (BFI).

#### Determination of the cut-off value

The BFI was determined by use of three different formulas. With the first formula [21], BFI = AB-CW, where AB represents the optical density at 595 nm (OD_595nm_) of a well containing stained attached bacteria and CW, the OD_595nm_ of the stained control wells containing bacteria-free medium only, here supplemented with BHI. With the second formula [22], BFI = AB/CW. With the third formula [23], BFI = (AB-CW)/G, where G is the OD_595nm_ of bacterial growth control. The assays were performed in duplicate. Finally, results were studied by terciles to classify biofilm production semi-quantitatively in three categories for each formula: strong production (S), moderate production (M) and absence of production (N) according to the cut-off values proposed by Naves *et al.*[24]. Because Naves *et al.*[24] showed that biofilm formation was strongly modulated by culture conditions, environmental factors and methodology, we combined the three BFI formulas to overcome above factors.

#### Statistical analysis

Double data entry was performed with use of the software 4D v 12. Univariate analysis and multivariate logistic regression models and estimation of odds ratios (ORs) and 95% confidence intervals [CI] involved use of Stata SE v11.2 (Stata Corp., College Station, TX, USA). The Pearson chi-square test was used to compare groups. Potential risk factors were identified by univariate analysis (*p* <0.25) and applied in multivariate logistic regression models. Factors considered were history of AOM, conjunctivitis, associated carriage of *S. pneumoniae* and BLNAR strains. P < 0.05 was considered statistically significant.

## Results

Among the 225 selected strains, 9 were not found in the collection of frozen samples and therefore could not be included. So, we analyzed the remaining 216 strains. All strains were identified by MALDI-TOF MS. When the result was not sufficiently discriminative between *H. influenzae* and *H. haemolyticus*, the identification of *H. influenzae* was confirmed by sequencing *sodA*_int_, *recA* and *fucK* genes [25].

Among the 171 isolates in NP samples, only 2 (1.1%) were serotype b *H. influenzae*. All of the 45 strains isolated in MEF were non-typable strains.

Among the 216 strains, 37 produced β-lactamase (17.1%), 32 were BLNAR (14.8%) and 7 showed both mechanisms of resistance (3.2%).

*1) Comparison of the three methods to calculate BFI*

Among the 216 studied strains, 162 (75%) were classified in the same category of biofilm production (strong, moderate or absence) by the three formulas. For 54 strains, results were discordant. However, in each case, results were concordant with two of the three formulas. Therefore, these 54 strains were classified by agreement in two of the three formulas. Finally, 47/216 (21.7%) strains of *H. influenzae* were classified as strong producers of biofilm and 59/216 (27.3%) as moderate producers; 110/216 (51%) did not produce biofilm. In total, 106 (49%) strains produced biofilm.

*2) Relationship between pathology and biofilm production*

To describe the effect of biofilm production by pathology, results were combined for two categories: strains producing biofilm (moderate or strong production) and strains not producing biofilm (negative).

In the control group of healthy children (NP group 1), 60.5% *H. influenzae* strains produced biofilm (Table 1).

**Table 1:** **Association of pathologic group and biofilm production by isolated *H. influenzae *** **strains**

**Groups**	**Level of biofilm production**				Comparison between groups *p value OR [95% CI]			
	**Negative**	**Moderate**	**Strong**	**Moderate + Strong**	**Reference vs Group 1**	**Reference vs Group 2**	**Reference vs Group 3**	**Reference vs Group 4**
Group MEF** n = 41	24 (58.5%)	8 (19.5%)	9 (22%)	17 (40.5%)	0.083	0.19	0.16	0.32
AOM treatment failure					2.16 [0.90;5.16]	1.78 [0.75;4.24]	1.86 [0.79;4.40]	0.64 [0.26;1.55]
NP group 1*** n = 43	17 (39.5%)	11 (25.6%)	15 (34.9%)	26 (60.5%)		0.66	0.73	0.006
Control (healthy children)						1.21 [0.51;2.86]	1.16 [0.49;2.73]	3.39 [1.41;8.15]
NP group 2*** n = 43	19 (44.2%)	18 (41.9%)	6 (13.9%)	24 (55.8%)			0.93	0.02
First AOM without conjunctivitis							0.96 [0.41;2.24]	2.8 [1.17;6.69]
NP group 3*** n = 45	19 (43.2%)	13 (29.5%)	12 (27.3%)	25 (56.8%)				0.015
Recurrent AOM without conjunctivitis								0.34 [0.14;0.82]
NP group 4*** n = 44	31 (68.9%)	9 (20%)	5 (11.1%)	14 (31.1.5%)				
AOM with conjunctivitis								

OR, odds ratio; 95% CI, 95% confidence interval.

*Risk for carrying (moderate + strong) an *H. influenzae* strain producing biofilm.

**Strains isolated from MEF.

***Strains isolated from the nasopharynx.

Considering the influence of biofilm production on AOM recurrence, we found no difference between strains in NP during a first AOM episode or during recurrent AOM in the absence of conjunctivitis (NP group 2 *vs* NP group 3, 55.8% *vs* 56.8%, respectively, *p* = 0.93, OR = 0.96 [95% CI, 0.41; 2.24]) (Table 1).

We found no significant difference in biofilm production from strains in MEF (children with AOM treatment failure) and strains in NP samples (NP groups 1, 2, 3 and 4): 40.5% *vs* 60.5% (*p* = 0.083, OR = 2.16 [0.90;5.16]), 55.8% (*p* = 0.19, 1.78 [0.75;4.24]), 56.8% (*p* = 0.16, 1.86 [0.79;4.40]) and 31.1% (*p* = 0.32, 0.64 [0.26;1.55]), respectively.

However, *H. influenzae* strains in NP samples from children with both AOM and conjunctivitis (NP group 4) produced significantly less biofilm (68.9% non-producer) than strains from NP groups 1, 2 and 3 (*p* = 0.006 and OR = 3.39 [1.41;8.15], *p* = 0.0; 2.8 [1.17;6.69], *p* = 0.015; and 0.34 [0.14;0.82], respectively). Biofilm production did not significantly differ between strains in MEF and in NP samples from children with AOM and conjunctivitis. For most of the comparisons, the number of patients was too small and the differences between groups too low to demonstrate significant differences.

Finally, biofilm production did not significantly differ between NP group 1 strains (healthy children, 60.5%) and those from children with a first or recurrent AOM without conjunctivitis, NP groups 2 (55.8%, *p* = 0.66, OR = 1.21 [0.51; 2.86]) and 3 (56.8%, *p* = 0.73, 1.16 [0.49; 2.73).

*3) Risk factors for carriage of H. influenzae producing biofilm*

We examined associations between risk factors for *H. influenzae* carriage and biofilm production by univariate analysis. Age less or greater than 12 months old as well as type of daycare (daycare center, home or nurse) were not associated with carriage of a strain producing biofilm (*p* = 0.36 and 0.61, respectively) (Table 2).

**Table 2:** **Risk factors for carriage of *H. influenzae *** **associated with biofilm production**

	**No. of strains (%)**	Biofilm non-producer* (n = 110)	Biofilm producer** (n = 106)	***P value***	**OR**
**Age: n =216**					
< 12 months	79 (36.6%)	37 (46.8%)	42 (53.2%)	0.361	
> 12 months	137 (63.4%)	73 (53.3%)	64 (46.72%)		
**Types of daycare: n = 216**					
Daycare center	117 (54.2%)	61 (52.1%)	56 (47.9%)	0.616	
Home	49 (22.7%)	22 (44.9%)	27 (55.1%)		
Nurse	50 (23.1%)	27 (54%)	23 (46%)		
**Association with *Streptococcus pneumoniae*: n = 216**					
Yes	97 (44.9%)	43 (44.3%)	54 (55.7%)	0.08	
No	119 (55.1%)	67 ( 56.3%)	52 (43.7%)		
**β-lactamase production: n = 216**					
Yes	37 (17.1%)	21 (56.80%)	16 (43.20%)	0.436	
No	179 (82.9%)	89 (49.72%)	90 (50.28%)		
**Penicillin-binding protein modification: resistance to amoxicillin: n =216**					
Yes	32 (14.8%)	22 (68.75%)	10 (31.25%)	0.029	2.4 [1.08;5.35]
No	184 (85.2%)	88 (47.83%)	96 (52.17%)		
**History of AOM: n =172**					
Yes	104 (60.5%)	54 (51.9%)	50 (48.1%)	0.168	
No	68 (39.5%)	28 (41.2%)	40 (58.8%)		
**Associated conjunctivitis: n = 214**					
Yes	54 (25.2%)	39 (72.2%)	15 (27.8%)	<0.001	3.3 [1.66;6.38]
No	160 (74.8%)	71 (44.4%)	89 (55.6%)		
**Previous antimicrobial therapy (<3 months): n = 210**					
None	91 (43.3%)	43 (47.2%)	48 (52.8%)	0.716	
Amoxicillin	70 (33.3%)	37 (52.9%)	33 (47.1%)		
3GC***	49 (23.4%)	26 (53.1%)	23 (46.9%)		
**Pneumococcal vaccination: n = 216**					
Yes	211 (97.7%)	106 (50.2%)	105 (49.8%)	0.188	
No	5 (2.3%)	4 (80.0%)	1 (20.0%)		

*No. of strains not producing biofilm.

**No. of strains producing biofilm.

***3GC: third-generation cephalosporins.

*H. influenzae* was often found with *S. pneumoniae* (44.9% of cases)*.* Although *H. influenzae* produced more biofilm with than without *S. pneumoniae* cohabitation (OR = 1.6 CI [0.94; 2.78]), the difference was not significant (55.7% with *vs* 43.7% without cohabitation, *p* = 0.08). Antibiotic treatment, amoxicillin or third-generation cephalosporin taken in the previous 3 months was not a risk factor for carrying an *H. influenzae* strain producing biofilm, *p* = 0.71 (Table 2).

Two factors were significantly associated with low biofilm production. The first was ampicillin resistance of *H. influenzae* by modification of the penicillin-binding protein (PBP) (BLNAR strains) (31.2% producing biofilm by ampicillin-resistant strains *vs* 52.2% by ampicillin-susceptible strains, *p =* 0.029; OR = 2.4 CI [1.08; 5.35]) (Table 2). The resistance to ampicillin mediated by β-lactamase did not affect biofilm production. The second risk factor associated with decreased biofilm production was the presence of conjunctivitis-otitis syndrome. Indeed, strains causing otitis and conjunctivitis produced significantly less biofilm than those from children with otitis without conjunctivitis: 27.8% *vs* 55.6%, *p* <0.001 (OR = 3.3, CI [1.66; 6.38]).

4) ***Multivariate analysis***

We examined predictors of low biofilm production by *H. influenzae* strains by multivariate analysis of past history of AOM, presence of conjunctivitis, co-colonization with *S. pneumoniae* and PBP modification. Only presence of conjunctivitis was significantly associated with low biofilm production (*p* = 0.001, OR = 0.3, CI [0.16-0.60]). The OR was not adjusted.

## Discussion

In this study, we examined whether *H. influenzae* strains in MEF from children with AOM were high biofilm producers, causing AOM treatment failure. We found conjunctivitis-otitis syndrome significantly associated with low biofilm production by *H. influenzae* strains (27.8% production with the syndrome *vs* 55.6% without). This association was not previously described. In the absence of conjunctivitis, biofilm production did not significantly differ between strains from children with a first, cured AOM and those with recurrent AOM.

One limitation of the study is the small number of patients in each group which does not allow for determining a significant difference in biofilm production by *H. influenzae* between strains from healthy controls and the MEF group (children with AOM treatment failure).

A second limitation is to compare strains from the MEF and NP samples. Some argue that strains in NP samples are not responsible for AOM. In a prospective study, Bingen *et al.* showed by pulsed-field electrophoresis that in conjunctivitis-otitis syndrome, *H. influenzae* strains from MEF and conjunctivitis were identical for the same child [26]. These data are reinforced by the fact that NT-*Hi* strains were not clonal and constituted a considerably diverse population. Van Dongen *et al*. [27], in a systematic review of the literature on the concordance between strains isolated from nasopharyngeal and MEF samples, found that *H. influenzae* strains were concordant in 80% of the cases.

AOM is the most common bacterial infection in children with *H. influenzae* and *S. pneumoniae* as the major causative agents. *H. influenzae* is often found in samples from children with recurrent AOM or AOM treatment failure [4]. A recent hypothesis was the ability of *H. influenzae* to produce biofilm and thus escape the antimicrobial treatment [11]. Bacterial growth in a self-produced ECM, preventing the action of antibiotics and an efficient host immune system, can explain the chronic nature of various infections. However, this hypothesis is still controversial [15]. Recently, Swords *et al.*[28] discounted the relevance of NT-*Hi* biofilms in disease. Langereis *et al.*[29] suggested that NT-*Hi* can lie in biofilm during both colonization and infection. The definition of a biofilm is still being discussed. Toretta *et al.*[30] reported greater biofilm production from NT-*Hi* from children with AOM than from healthy children. However, this study included only 12 AOM strains and only one healthy control strain.

We found significantly low biofilm production in strains with than without modified PBP (OR = 2.4 (CI [1.08; 5.35]). One hypothesis for this phenomenon is that resistance to amoxicillin and amoxicillin/clavulanic acid may promote the planktonic state. As well, biofilm production could be considered a real mechanism of resistance, and ß-lactamase production could obviate any resistance mechanism. These data need to be confirmed and explored in further studies.

The ECM secreted by *H. influenzae* is composed of lipo-oligosaccharides, proteases and adhesins but not a specific biofilm protein [31]. We studied biofilm production by *H. influenzae* stains with the "biofilm ring test" (Biofilm Control, Saint Beauzire, France) [32]. In the absence of a standardized method for *H. influenzae* (mainly due to the broth medium used and the need for 5% CO_2_ atmosphere), non-reproducible results were obtained. Therefore, we evaluated biofilm production of *H. influenzae* by the modified microtiter plate assay with CV staining [18]. However, this method still has the drawback of possibly removing the established biofilm when wells are washed. As well, CV as a cationic dye can stain a biofilm complex structure but also a group of living and dead bacteria. A limitation of our study is the lack of direct evaluation of biofilm on biological samples with alternative methods such as confocal microscopy after live/dead staining [33,34]. This method cannot be used with swabs and cannot be applied retrospectively. Furthermore, although swabs were in a transport medium, we cannot exclude that the delay in bacterial culture influenced the results. Therefore, we used three formulas to calculate biofilm production, and quantifying bacterial biofilm.

Rayner *et al.* hypothesized that *H. influenzae* could not be isolated from MEF cultures because the biofilm production disallowed cultivation [34]. AOM bacterial etiology was suspected because bacterial genetic material was found by RT-PCR. This hypothesis can explain the lower rate of biofilm-producing strains in the MEF group (treatment failure). Indeed, in this group, a double population may exist and the isolated strain was not the one producing biofilm; the responsive one may have been inside a biofilm structure and could not grow on agar plates.

In another study of 62 children with AOM, 84.3% of strains produced biofilm [33]. Nevertheless, this study lacked a control group. Clinical follow-up showed no significant difference in biofilm production between strains isolated from children with cured AOM and from patients with AOM treatment failure after 14 days of appropriate antibiotic therapy (78.6% vs 90%).

The co-localization of NT-*Hi* and *S. pneumoniae* suggests a synergistic interaction between the two organisms. To explain AOM treatment failure in the chinchilla middle ear, Weimer *et al.* suggested that *H. influenzae* and *S. pneumoniae* cohabitation may promote biofilm production of *S. pneumoniae*[35]. However, Tikhomirova A. *et al.*[36] questioned whether the relation between *H. influenzae* and *S. pneumoniae* is competitive or cooperative. We found no difference in biofilm production with *H.influenzae* alone or combined with *S. pneumoniae*.

Slinger *et al.* showed that MICs of *H. influenzae* for various antibiotics (amoxicillin, amoxicillin/clavulanic acid, ciprofloxacin) were higher when bacteria are living in the biofilm than in the planktonic state [37]. As well, Starner *et al.* showed that a low concentration of various antibiotics stimulated biofilm production by *H. influenzae*[38]. We determined whether prior administration of antibiotics was a risk factor of carrying a biofilm-producing strain. Indeed, according to the biofilm definition, bacteria living in an ECM resist the action of antibiotics. Conversely, Ehrlich *et al.* showed that after inoculation of *H. influenzae* into the middle ear of chinchillas, strains produced an ECM whose amount increased with time [39]. Then, after an administration of antibiotics 96 hr, bacterial biofilm disappeared, whereas, by definition, it was expected to be resistant to antibiotics. We found no significant difference in biofilm production among strains from children with and without antibiotics treatment (amoxicillin/clavulanic acid or 3GC) in the previous 3 months. We did not confirm that prior administration of antibiotics affected biofilm production by *H. influenzae*.

Finally, we observed two kinds of strains: 1) strains with a high production of biofilm (55.8% to 60.5%) in NP samples from healthy children and from AOM children without conjunctivitis and 2) strains with a lower production of biofilm (40.5% and 31.15%) in MEF or NP samples from children with AOM and conjunctivitis, respectively.

Recently, Sanchez *et al.*[40] examined whether *S. pneumoniae* in biofilms was virulent and contributed to invasive pneumococcal disease (IPD) development. The authors suggested that biofilms did not directly contribute to development of IPD and may instead confer a quiescent mode of growth during colonization. They challenged mice with equal colony-forming units of biofilm and planktonic pneumococci and determined that biofilm bacteria production was highly attenuated in invasive disease but not NP colonization.

Finally, we observed two kinds of strains: 1) strains with a high production of biofilm (55.8% to 60.5%) in NP samples from healthy children and from AOM children without conjunctivitis and 2) strains with a lower production of biofilm (40.5% and 31.15%) in MEF or NP samples from children with AOM and conjunctivitis, respectively.

## Conclusions

This study emphasizes the frequency of biofilm production by *H. influenzae* and also the complexity of the phenomenon, because 49% of the strains produced biofilm. We did not find increased biofilm production in strains isolated from children with AOM treatment failure or recurrent AOM. *H. influenzae* involved in the conjunctivitis-otitis syndrome showed significantly low biofilm production. We observed the same low production in strains with PBP modification, conferring resistance to ampicillin, as compared with susceptible strains. These observations require study of a larger number of strains to confirm these results and explore other directions. To our knowledge, this is the first study evaluating the biofilm production by *H. influenzae* in NP samples from healthy children. With 49% of the strains producing biofilm, growing inside a biofilm may constitute a natural mode of growth for the bacteria, especially in the upper airways of children.

## Competing interests

The authors declare that they have no competing interests.

## Authors’ contributions

AM performed experiments and wrote the manuscript. JR led the study and write the manuscript. SB and CL designed the study and performed the statistical analysis. SB and EV provided the strains. RC write the manuscript. All authors read and approved the final manuscript.

## Pre-publication history

The pre-publication history for this paper can be accessed here:

http://www.biomedcentral.com/1471-2334/14/400/prepub
